# Analysis of the treatment outcome of duodenal varices: A retrospective case series of 15 patients from a single institution

**DOI:** 10.1002/deo2.70119

**Published:** 2025-04-16

**Authors:** Yuri Mitamura, Eisuke Murakami, Ko Hashimoto, Tomoaki Emori, Aiko Tanaka, Yusuke Tanaka, Keiichi Hiraoka, Yuki Shirane, Masanari Kosaka, Yusuke Johira, Ryoichi Miura, Serami Murakami, Kenji Yamaoka, Yasutoshi Fujii, Shinsuke Uchikawa, Hatsue Fujino, Atsushi Ono, Tomokazu Kawaoka, Daiki Miki, Clair Nelson Hayes, Masataka Tsuge, Keigo Chosa, Kazuo Awai, Shiro Oka

**Affiliations:** ^1^ Department of Gastroenterology Graduate School of Life Science Institute of Biomedical & Health Science Hiroshima University Hiroshima Japan; ^2^ Liver Disease Center Hiroshima University Hospital Hiroshima Japan; ^3^ Department of Diagnostic Radiology Graduate School of Biomedical and Health Sciences Hiroshima University Hiroshima Japan

**Keywords:** balloon‐occluded retrograde transvenous obliteration, duodenal varices, endoscopic variceal ligation therapy, percutaneous transhepatic variceal obliteration, portal hypertension

## Abstract

**Background & aims:**

Duodenal varices (DVs) are a rare type of ectopic varices occurring in portal hypertension, for which no standardized treatment strategy has been established. This retrospective study analyzed the outcomes of DV treatments in 15 patients.

**Material and methods:**

All enrolled patients with DVs were treated at a single institution Hospital between 2011 and 2022. The treatment procedure and outcome were analyzed retrospectively.

**Results:**

Six patients presented with hemorrhagic DVs. Endoscopic variceal ligation was used for initial hemostasis in five bleeding cases. Balloon‐occluded retrograde transvenous obliteration was the initial treatment in nine cases, achieving curative obliteration in eight cases. Percutaneous transhepatic variceal obliteration was performed as the initial treatment in three cases for which balloon‐occluded retrograde transvenous obliteration was difficult to perform for anatomical reasons, and all cases achieved curative obliterations. Splenectomy was performed as the initial treatment in three patients due to complicating gastroesophageal varices. DVs recurred in two cases with splenectomy after approximately 1 year, but balloon‐occluded retrograde transvenous obliteration and percutaneous transhepatic variceal obliteration were curatively applied in each case, and no recurrence has been observed since then. Gastroesophageal varices aggravated after the initial DV treatment in eight of the 15 cases during the observation period, and the cumulative aggravating rate was 58.1% at 4 years.

**Conclusion:**

All 15 cases with DVs were preferably controlled by selecting appropriate treatment based on individual hemodynamics of varices. Because of the relatively high rate of aggravation of gastroesophageal varices, careful long‐term follow‐up may be important for the treatment of DVs.

## INTRODUCTION

Duodenal varices (DVs) are a relatively rare type of collateral between the portal vein and systemic circulation that occurs in patients with portal hypertension.^1^ Collaterals developing at sites other than esophageal or gastric varices have been broadly termed ectopic varices,^2^ and DVs are reported to be the second most common type of ectopic varices after rectum varices.^3^ Most DVs are located in the descending to transverse parts of the duodenum. DVs are reported to be the most common site of ectopic variceal bleeding, and bleeding of DVs has been reported to be a fatal complication with a mortality rate as high as 40%.^4^ However, the management of bleeding from DVs can be challenging due to the difficulty of treatment.^5^ It is difficult to determine the best treatment strategy for DVs because the hemodynamics of DVs tend to vary by individual.^6^ Most studies involve small series and case reports with no randomized therapeutic trials and may include treatment with endoscopic ligation or sclerotherapy, interventional embolization of the feeding vessels or portosystemic stent shunts, and surgery.^7^, ^8^, ^9^, ^10^, ^11^, ^12^, ^13^, ^14^ Although no fixed treatment strategy has been fully accepted for DVs, prompt hemostatic treatment and preventive radical treatment are necessary for treating DVs via selecting appropriate treatment based on the individual hemodynamics of DVs. In this retrospective cohort study, the treatment procedure and prolonged outcome were analyzed in a consecutive case series of 15 patients with DVs who were treated by endoscopic variceal ligation therapy (EVL), balloon‐occluded retrograde transvenous obliteration (B‐RTO), and percutaneous transhepatic variceal obliteration (PTO) at Hiroshima University Hospital between 2011 and 2022.

## METHODS

At Hiroshima University Hospital, 15 patients were diagnosed with DVs between March 2011 and April 2022.

All enrolled patients underwent esophagogastroduodenoscopy (EGD) to confirm the diagnosis of DVs with or without DVs bleeding, and the co‐existence of esophagogastric varices was determined according to the guidelines of the Japan Society for Portal Hypertension.^15^ The form (F) of the varices was classified as small straight (F1), enlarged tortuous (F2), and large coiled‐shaped (F3). The red color (RC) sign was also classified based on the criteria of the Japanese Society for Portal Hypertension and Esophageal Varices ^15^ DVs with bleeding, F2‐3, and those with a tendency to enlarge were indicated for treatment.

### Treatment procedure of DVs

All patients received esophagogastroduodenoscopy and enhanced computed tomography (CT) scan prior to the treatment to estimate the hemodynamics of each DV by identifying the main feeder vein and the drainage vein anatomically. The device used for the endoscopic treatment was a single‐channel forward‐viewing endoscope (Olympus models Q260J, H260, or H290; Olympus Optical Co.). Hemorrhagic DVs were immediately treated by EVL at the bleeding point of the DVs for primary hemostasis. EVL was performed using the established procedure using an endoscopic variceal ligation device (MD48709U; Sumitomo Bakelite) as previously reported. All endoscopic treatments were carried out by two or more experienced endoscopists.

The indication B‐RTO was secondarily considered by estimating the hemodynamics of each DV by identifying the main feeder vein and the drainage vein. In bleeding cases, those with portosystemic shunts identified during the CT scan were indications for B‐RTO after endoscopic primary hemostasis. B‐RTO was performed using the established procedure, as previously reported.^16^, ^17^, ^18^ To perform the B‐RTO procedure, a 5‐Fr balloon catheter (Selecon MP catheter; Terumo Clinical Supply) was inserted into the portosystemic shunt through the inferior vena cava via the right femoral or right jugular vein under local anesthesia. Balloon‐occluded retrograde venography was performed to determine the hemodynamics of the DVs and collateral veins after the blockade of outflow vessels from the shunt by balloon occlusion. After the DVs were visualized by retrograde venography under fluoroscopy, the B‐RTO procedure was performed using sclerosing agents, commonly 10% ethanolamine oleate (Oldamin; Takeda Pharmaceutical) mixed with the same volume of the non‐ionic contrast medium iopamidol (Iopamiron 300; Bayer Healthcare), with 5% ethanolamine oleate mixed with iopamidol under balloon occlusion. Representative clinical cases will be shown (Supporting Information).

The indication PTO was thirdly considered when the hemodynamics of each DV were considered inappropriate or refractory for B‐RTO after identifying excess bold or small‐diameter drainage veins or identifying multiple outflow vessels by balloon occlusion with the absence of ascites for intrahepatic approach from the abdominal wall. PTO was also performed using the established procedure. After 10 mL of 1% lidocaine (Xylocaine; AstraZeneca K.K.) was injected into the peritoneum along the puncture line, percutaneous transhepatic puncture of the intrahepatic branch of the portal vein was performed using a 21‐gauge needle under sonographic guidance. A 5‐French gauge sheath catheter was then introduced into the portal vein using a two‐step approach.^19^ Direct intrahepatic portography was performed to identify the feeding and draining veins of the DVs. After the DVs were visualized by direct portography under fluoroscopy, the PTO procedure was performed using microcoil or sclerosing agents using a mixture of N‐butyl‐2‐cyanoacrylate (Histoacryl; B. Braun) and contrast agents of Lipiodol (Laboratoire Guerbet) in a volume/volume ratio from 1:1 to 1:3 which were based on the physicians’ decisions. All patients underwent contrast‐enhanced CT scanning and EGD one week after B‐RTO or PTO to clarify the technical success, which was estimated by observation of low attenuation, disappearance of enhancement on CT, and reduction in size of the DVs by EGD.

### Follow‐up after the treatment of DVs

The endoscopic findings of DVs and gastro‐esophageal varices were evaluated according to the classification system of the Japanese Society for Portal Hypertension and Esophageal Varices.^15^ Endoscopic examinations were performed every 6 months or 1 year after the treatment of DVs to follow‐up DVs and gastro‐esophageal varices. Worsening of the F and RC signs compared to baseline findings on follow‐up EGD was defined as aggravation of DVs. Gastroesophageal varices were defined as F2 or higher and positive RC signs as worsening. Aggravated varices were treated by endoscopic injection sclerotherapy (EIS), endoscopic variceal ligation (EVL), and interventional embolization including B‐RTO or PTO when the attending physician determined that the varices required treatment.

### Clinical data and statistical analysis

The laboratory or physical data were collected before the treatment of DVs, and survival and gastro‐esophageal varices aggravation were examined as in our previous report.^18^ The cumulative survival and gastro‐esophageal varices aggravation rates were determined using the Kaplan–Meier method The data were statistically analyzed in July 2024. Statistical analyses were performed using IBM SPSS Statistics for Windows (version 22.0; IBM Corp.).

## RESULTS

### Patients

The median age was 68 years old, and nine males and six females were enrolled. DVs were observed at the descending region of the duodenum in 14 patients (93.3%) and at the transverse region of the duodenum in one patient. Six cases (40%) had hemorrhagic DVs. All patients had underlying cirrhosis, with the cause due to alcohol in six cases, hepatitis C virus in six, hepatitis B virus in one, autoimmune hepatitis in one, and unknown causes in one case. Liver function was classified as Child‐Pugh class A in six cases (40%), class B in eight cases (53.3%), and class C in one case. Patient characteristics are summarized in Table 1.

**TABLE 1:** Patient characteristics

No.	Age	Gender	Etiology	Bleeding case	Location within duodenum	Prior hemostatic treatment	Initial treatment	Secondary treatment	Feeding vein	Drainage vein	HVPG (mmHg)	Coexisting varices	Child‐Pugh class	HCC
1	85	F	HCV	Yes	Descending	EVL	B‐RTO	–	SMV	RAV	–	EV	B	+
2	53	M	HCV	No	Descending	None	Splenectomy	–	SMV	RTV	–	EV/GV	A	+
3	75	F	HCV	Yes	Descending	EVL	B‐RTO	PTO	SMV	ROV	11	–	B	
4	70	M	Alc	No	Descending	None	B‐RTO	–	SMV	RTV	16	EV	A	
5	70	M	Alc	No	Descending	None	PTO	–	SMV	RTV	4	EV/GV	A	+
6	64	F	SLD	No	Descending	None	Splenectomy	PTO	SMV	IVC	17	EV	A	
7	66	F	Alc	No	Descending	None	PTO	–	SMV	ROV	22	EV	B	
8	74	M	HBV	No	Descending	None	Splenectomy	B‐RTO	SMV	RAV	12	–	B	
9	44	M	HCV	Yes	Horizontal	EVL	PTO	–	SMV	RTV	–	EV	C	
10	73	F	HCV	No	Descending	None	B‐RTO	–	SMV	IVC	11	–	A	
11	51	F	Alc	Yes	Descending	None	B‐RTO	–	SMV	ROV	17	EV/GV	B	
12	56	M	Alc	No	Descending	None	B‐RTO	–	SMV	RTV	15	EV/GV	B	
13	46	M	Alc	Yes	Descending	EVL	B‐RTO	–	SMV	IVC	–	EV	B	
14	73	M	AIH	No	Descending	None	B‐RTO	–	SMV	LIV	14	–	A	+
15	64	M	HCV	Yes	Descending	EVL	B‐RTO	–	PV	RAV	–	–	B	

Abbreviations: AIH, autoimmune hepatitis; Alc, alcohol; EV, esophageal varices; EVL, endoscopic variceal ligation; GV, gastric varices; HBV, hepatitis B virus; HCC, hepatocellular carcinoma; HCV, hepatitis C virus; HVPG, hepatic venous pressure gradient; IVC, inferior vena cava; LIV, left iliac vein; PV, portal vein; RAV, right adrenal vein; ROV, right ovarian vein; RTV, right testicular vein; SLD, steatotic liver disease; SMV, superior mesenteric vein.

### Hemodynamics of DVs

In a comparative study of the bleeding and non‐bleeding groups, there were no significant differences between the two groups in terms of site of presence, F factor, or presence of RC sign (Fisher's exact test); RC sign was not present in all patients. Throughout the prior EGD examinations, co‐existing esophageal varices were present in 10 cases (66.7%) and gastric varices in four cases (26.7%) at the time of diagnosis of DVs. Five out of six patients with hemorrhagic DVs were initially treated by EVL at the bleeding point of the DVs for prior hemostasis. The main feeder veins of DVs were branches of the superior mesenteric vein in 14 cases (93.3%) and the main trunk of the portal vein in one case. The drainage vein varied, ranging from the right adrenal vein in three cases, the right gonadal (testicular or ovarian) vein in eight cases, the inferior vena cava in three cases, and the left iliac vein in one case. No patients were pointed out of the existence of portal vein thrombosis. The baseline clinical characteristics of the fifteen patients are summarized in Table 1.

### Treatment Procedure of DVs

**FIGURE 1:**
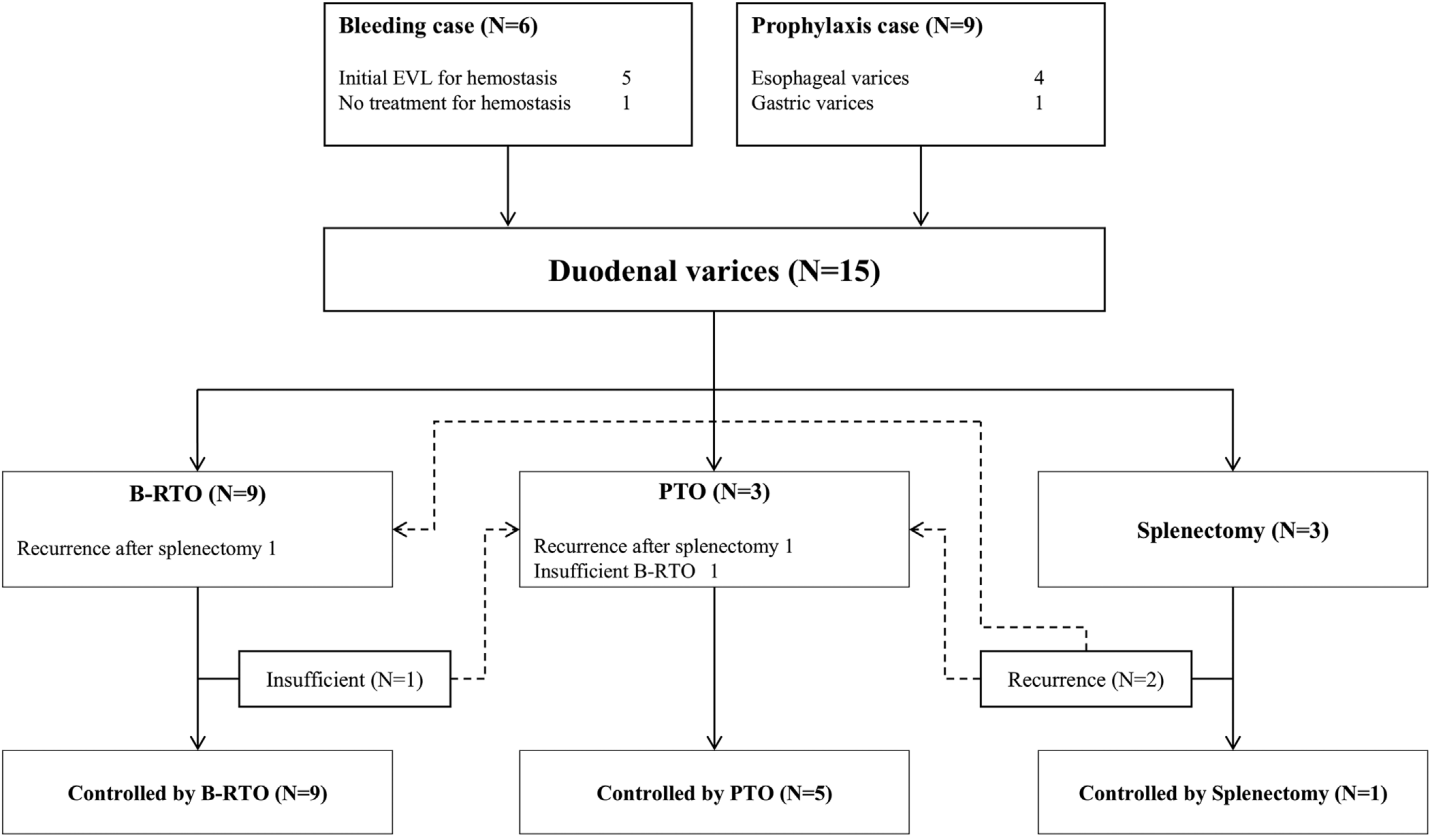
Treatment procedure of duodenal varices. Five of the six bleeding duodenal varice (DV) cases were treated with endoscopic variceal ligation (EVL) therapy for initial hemostasis. Balloon‐occluded retrograde transvenous obliteration (B‐RTO) was performed as the initial treatment in eight cases, and curative obliterations were achieved in seven cases. In another case for which B‐RTO was insufficient, percutaneous transhepatic variceal obliteration (PTO) was successfully administered on the same day. PTO was performed as the initial treatment in three cases in which B‐RTO was difficult to perform for anatomical reasons, and all cases achieved curative obliterations. Splenectomy was performed as the initial treatment in three patients because of complicating gastroesophageal varices. DVs recurred in two cases with splenectomy after approximately 1 year, and B‐RTO or PTO was curatively applied in each case.

EGD diagnosed DVs before treatment in the descending region of the duodenum in 14 of the 15 cases, which was followed by endoscopic variceal ligation therapy in five cases of variceal bleeding for initial hemostasis. In one case, EVL was not performed because the patient had spontaneous hemostasis at the time of observation and no erosions or other bleeding spots were noted. B‐RTO was performed as the initial treatment in 9 cases, and curative obliterations were achieved in eight cases. In another case in which B‐RTO was insufficient, PTO was successfully administered on the same day. PTO was performed as the initial treatment in three cases for which B‐RTO was deemed difficult to perform due to anatomical considerations, and all cases achieved curative obliterations. Splenectomy was performed as the initial treatment in three patients due to the presence of gastroesophageal varices.^20^ DVs recurred in two cases with splenectomy after approximately 1 year, and B‐RTO or PTO was curatively applied in each case. All cases with DVs, both hemorrhage and prophylaxis cases, were controlled by selecting appropriate treatment and additional treatment with B‐RTO, PTO, and splenectomy and they progressed without recurrence (Figure 1).

### Adverse events and long‐term outcomes

**FIGURE 2:**
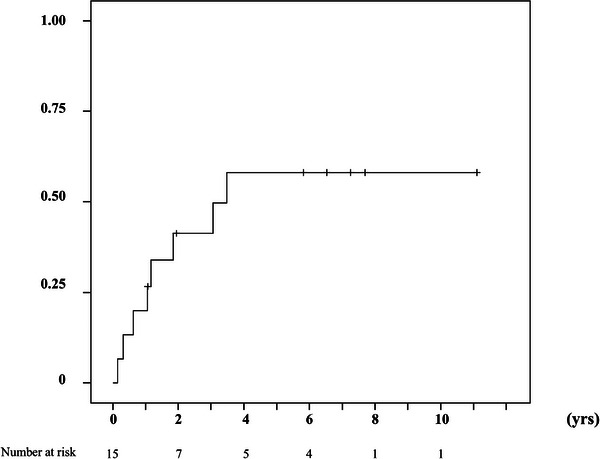
Cumulative aggravation rate of gastro‐esophageal varices after the treatment of duodenal varices. Gastro‐esophageal varices aggravated after the treatment of duodenal varices in eight out of 15 patients. The cumulative aggravation rate of gastro‐esophageal varices 1, 2, and 4 years after treatment was 26.7%, 41.3%, and 58.1%, respectively.

Complications after treatment of duodenal varices included fever in eight cases, ascites in six cases, portal vein thrombus in five cases (four cases in PTO and one case in splenectomy), and coil migration in one case. The patients with complications all improved following medical therapy or careful observation. Gastroesophageal varices aggravated after the initial treatments of DVs in eight out of 15 cases (53.3%) during the observation period, and the cumulative varices aggravating rate was 26.7 % at 1 year and 58.1% at 4 years (Figure 2). No difference in the recurrence of DVs and gastroesophageal varices was observed between treatments (Fisher's exact test).

## DISCUSSION

Because duodenal varices are a type of ectopic varices, excluding esophagogastric varices, and are relatively rare, reported in only 0.4% of patients with portal hypertension,^21^ few papers have been demonstrating the treatment protocols of DVs except for this present study.

Particular attention also should be paid to possible hemodynamic changes following treatment of duodenal varices due to the influx of portal blood into various vessels. A standard treatment for duodenal varices has not yet been established, and various treatments are currently being explored. In general, treatment methods include endoscopic therapy, embolization using interventional radiology (IVR) such as PTO,^22^ Transjugular Intrahepatic Portosystemic Shunt^23^ (TIPS is not covered by insurance in Japan), and B‐RTO.^5^, ^24^ On the other hand, DVs are thought to pose a latent risk for rupturing and bleeding, and the blood flow of bleeding DVs has been reported to be massive and fatal, with a mortality rate as high as 40%.^4^ Therefore, prompt hemostatic treatment is essential. As endoscopic surgery is generally less invasive, quicker, and easier to perform compared to surgery or IVR, endoscopic treatment has been recommended as the primary hemostasis in cases of bleeding.^25^ As endoscopic EVL can cause large postoperative ulceration, rebleeding, and hemorrhagic shock if sufficient thrombus formation is not achieved, there are reports that EIS using cyanoacrylates is appropriate for duodenal varices.^11^, ^26^ However, duodenal varices, like gastric varices, are major portal shunts and have limited endoscopic treatment because blood flow is more rapid and abundant.^5^ In a recent study, varicography during IVR sometimes showed significant duodenal variceal blood flow even after endoscopic treatment.^5^ On this point, definitive IVR in addition to endoscopic treatment is considered necessary to achieve long‐term control.^5^ If IVR can be performed immediately after EVL, EVL, is thought to be a simple and quick treatment compared to EIS, and may be an alternate treatment option for DVs. EVL also allows time for patients to be transferred to a facility with IVR or other equipment. Although there were no cases in which EIS was performed in this study, EIS is an important treatment for duodenal varices and should be compared with EVL in the future.

**FIGURE 3:**
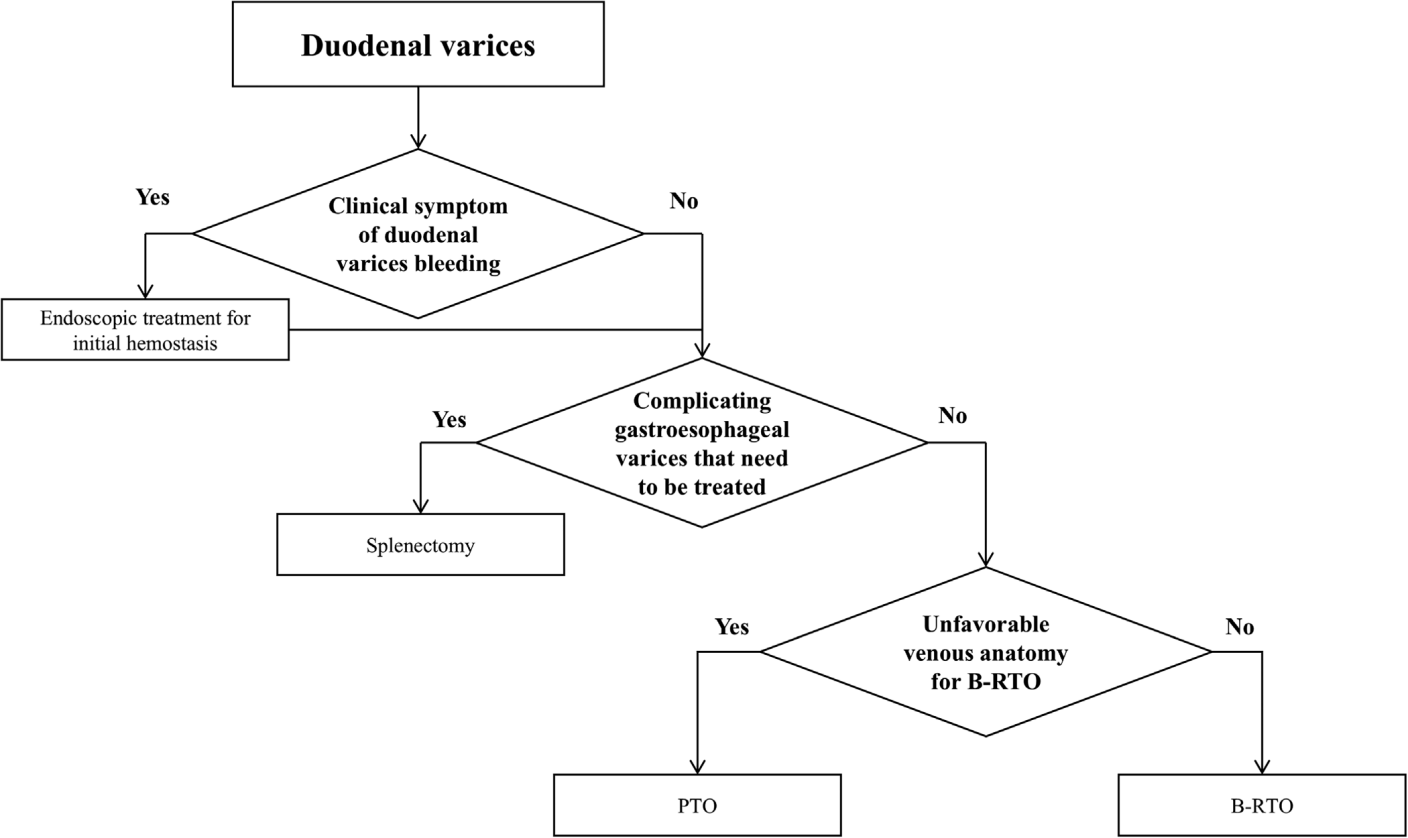
Therapeutic flowchart for duodenal varices. B‐RTO, balloon‐occluded retrograde transvenous obliteration; PTO, percutaneous transhepatic variceal obliteration.

Taking into account the advantages and disadvantages of the treatment of DVs as described above, our treatment protocol is shown in Figure 3. In the case of hemorrhagic DVs in which emergency hemostasis is necessary, EVL is attempted to achieve temporary hemostasis, and IVR treatment is considered immediately afterward to stabilize the hemodynamics of DVs to prevent re‐bleeding. If a retrograde route via the drainage vein to approach the portosystemic shunt to the DVs can be confirmed by a CT scan, B‐RTO is first considered to stabilize the hemodynamics of the DVs. If the drainage route is not confirmed, PTO is secondarily considered via the intrahepatic portal vein to approach the feeding side of the portosystemic shunt. For patients with stable circulation and without a history of DV bleeding as prophylactic cases, IVR is considered to apply because of its curative effect, which can reliably embolize the vessel through the blood supply route. In non‐urgent prophylactic cases with esophagogastric varices, hemoperitoneum, and splenectomy including inflow port‐systemic shunt treatment (such as the Hassab procedure) may be an optimal treatment option because there may be some risk of aggravation of esophagogastric varices after IVR.^27^ On the other hand, the possibility of DV recurrence should be kept in mind, as DVs recurred in two of the three cases after splenectomy, after which BRTO or PTO were successfully administered. Thus, all DVs might be controllable by combining multiple treatments such as endoscopic therapy, IVR, and splenectomy according to the anatomical conditions of each case. There may remain an alternate treatment option of IVR partial splenic embolization (PSE) and β‐blockers were reported to improve portal hypertension.^28^ PSE and β‐blockers are treatment options for portal hypertension, but there are no reports of their use in the treatment of duodenal varices.

Hepatic venous pressure gradient (HVPG) is a well‐established standard marker for diagnosing clinically significant portal hypertension. HVPG is usually 1–5 mmHg, but greater than 10 mmHg is associated with ascites and variceal progression, and greater than 12 mmHg with variceal bleeding.^29^, ^30^ In a recent study, the HVPG of 10 patients was measured during IVR, and HVPG elevation was observed in most of them; nine out of 10 patients had elevated HVPG greater than 10 mmHg, and seven had HVPG greater than 12 mmHg, which is considered to be clinically significant portal hypertension^29^, ^30^ (Table 1). Esophageal varices were also reported to aggravate after treatment of duodenal varices.^27^ In a recent study, gastroesophageal varices worsened in eight patients (53.3%) after initial treatment of duodenal varices at a relatively high rate. The cumulative rate of worsening gastroesophageal varices was 26.7%/year, and 58.1% in 4 years. After treatment for duodenal varices, recurrence of gastroesophageal varices should be noted, and regular endoscopic examination should be performed.

The present study has certain limitations, including limited sample size and single‐center data including only 15 patients from a single institution. Furthermore, there appeared to be some selection bias based on our study protocol, such as not conducting an EIS. However, our results may provide supportive data for future multi‐center large‐scale studies aimed at accurately identifying acceptable treatment protocols for DVs.

## CONCLUSION

All 15 cases with DVs were preferably controlled by selecting appropriate treatment with B‐RTO, PTO, and splenectomy based on individual hemodynamics of varices. On the other hand, as the rate of aggravation of gastroesophageal varices was relatively high, careful long‐term follow‐up is important for the treatment of DVs (Figure S1–S4).

## CONFLICT OF INTEREST STATEMENT

None.

## ETHICS STATEMENT

This study was conducted in accordance with the ethics principles of the Declaration of Helsinki and was approved by the Institutional Review Board of Hiroshima University. Written informed consent was obtained from the patient after a detailed explanation of the study procedure.

## CLINICAL TRIAL REGISTRATION

N/A

## Supporting information



Fig S1: A 56‐year‐old man with alcoholic cirrhosis was pointed out a duodenal varix in the descending part of the duodenum.

Fig S2: Contrast‐enhanced computed tomography scanning showed that a small branch of the superior mesenteric vein was the major feeding vein of the duodenal varices, and the right testicular vein was the drainage vein.

Fig S3: During the balloon‐occluded retrograde transvenous obliteration, multiple drainage veins were detected that required coil embolization.

Fig S4: The complete regression of the duodenal varices was confirmed after 7 months of the treatment.

## References

[1] Henry ZH , Caldwell SH . Management of bleeding ectopic varices. Tech Gastrointest Endosc 2017; 19: 101–107.

[2] Norton ID , Andrews JC , Kamath PS . Management of ectopic varices. Hepatology 1998; 28: 1154–1158. 9755256 10.1002/hep.510280434

[3] Watanabe N , Toyonaga A , Kojima S *et al.* Current status of ectopic varices in Japan: Results of a survey by the Japan Society for Portal Hypertension: Current status of ectopic varices in Japan. Hepatol Res 2010; 40: 763–776. 20649816 10.1111/j.1872-034X.2010.00690.x

[4] Khouqeer F , Morrow C , Jordan P . Duodenal varices as a cause of massive upper gastrointestinal bleeding. Surgery 1987; 102: 548–552. 3498234

[5] Kakizaki S , Toyoda M , Ichikawa T *et al.* Clinical characteristics and treatment for patients presenting with bleeding duodenal varices: Treatment for duodenal varices. Dig Endosc 2010; 22: 275–281. 21175479 10.1111/j.1443-1661.2010.01007.x

[6] Liu Y , Yang J , Wang J *et al.* Clinical characteristics and endoscopic treatment with cyanoacrylate injection in patients with duodenal varices. Scand J Gastroenterol 2009; 44: 1012–1016. 19513934 10.1080/00365520903030787

[7] Kochar N , Tripathi D , McAvoy NC , Ireland H , Redhead DN , Hayes PC . Bleeding ectopic varices in cirrhosis: The role of transjugular intrahepatic portosystemic stent shunts. Aliment Pharmacol Ther 2008; 28: 294–303. 19086235 10.1111/j.1365-2036.2008.03719.x

[8] Yoshida Y , Imai Y , Nishikawa M *et al.* Successful endoscopic injection sclerotherapy with N‐butyl‐2‐cyanoacrylate following the recurrence of bleeding soon after endoscopic ligation for ruptured duodenal varices. Am J Gastroenterol 1997; 92: 1227–1229. 9219810

[9] Bosch A , Marsano L , Varilek GW . Successful obliteration of duodenal varices after endoscopic ligation. Dig Dis Sci 2003; 48: 1809–1812. 14561006 10.1023/a:1025402411557

[10] Wang CS , Jeng LB , Chen MF . Duodenal variceal bleeding–successfully treated by mesocaval shunt after failure of sclerotherapy. Hepatogastroenterology 1995; 42: 59–61. 7782038

[11] Ota K , Shirai Z , Masuzaki T *et al.* Endoscopic injection sclerotherapy with n‐butyl‐2‐cyanoacrylate for ruptured duodenal varices. J Gastroenterol 1998; 33: 550–555. 9719241 10.1007/s005350050131

[12] Tazawa J , Sakai Y , Koizumi K *et al.* Endoscopic ligation for ruptured duodenal varices. Am J Gastroenterol 1995; 90: 677–678. 7717347

[13] Barbish AW , Ehrinpreis MN . Successful endoscopic injection sclerotherapy of a bleeding duodenal varix. Am J Gastroenterol 1993; 88: 90–92. 8420280

[14] Haruta I , Isobe Y , Ueno E *et al.* Balloon‐occluded retrograde transvenous obliteration (BRTO), a promising nonsurgical therapy for ectopic varices: A case report of successful treatment of duodenal varices by BRTO. Am J Gastroenterol 1996; 91: 2594–7. 8946993

[15] Tajiri T , Yoshida H , Obara K *et al.* General rules for recording endoscopic findings of esophagogastric varices (2nd edition): Endoscopy of esophagogastric varices. Dig Endosc 2010; 22: 1–9. 20078657 10.1111/j.1443-1661.2009.00929.x

[16] Hiraga N , Aikata H , Takaki S *et al.* The long‐term outcome of patients with bleeding gastric varices after balloon‐occluded retrograde transvenous obliteration. J Gastroenterol 2007; 42: 663–672. 17701130 10.1007/s00535-007-2077-1

[17] Naeshiro N , Aikata H , Kakizawa H *et al.* Long‐term outcome of patients with gastric varices treated by balloon‐occluded retrograde transvenous obliteration: Gastric varix with transvenous obliteration. J Gastroenterol Hepatol 2014; 29: 1035–1042. 24372807 10.1111/jgh.12508

[18] Shirane Y , Murakami E , Imamura M *et al.* Hepatic venous pressure gradient after balloon‐occluded retrograde transvenous obliteration and liver stiffness measurement predict the prognosis of patients with gastric varices. BMC Gastroenterol 2022; 22: 535. 36550416 10.1186/s12876-022-02616-z PMC9773455

[19] Ishikawa T , Imai M , Ko M *et al.* Percutaneous transhepatic obliteration and percutaneous transhepatic sclerotherapy for intractable hepatic encephalopathy and gastric varices improves the hepatic function reserve. Biomed Rep 2017; 6: 99–102. 28123716 10.3892/br.2016.811 PMC5244787

[20] Oshita K , Ohira M , Honmyo N *et al.* Treatment outcomes after splenectomy with gastric devascularization or balloon‐occluded retrograde transvenous obliteration for gastric varices: A propensity score‐weighted analysis from a single institution. J Gastroenterol 2020; 55: 877–887. 32533300 10.1007/s00535-020-01693-9 PMC7289714

[21] Hashizume M , Tanoue K , Ohta M *et al.* Vascular anatomy of duodenal varices: Angiographic and histopathological assessments. Am J Gastroenterol 1993; 88: 1942–1945. 8237946

[22] Menu Y , Gayet B , Nahum H . Bleeding duodenal varices: Diagnosis and treatment by percutaneous portography and transcatheter embolization. Gastrointest Radiol 1987; 12: 111–113. 3493935 10.1007/BF01885117

[23] Vidal V , Joly L , Perreault P , Bouchard L , Lafortune M , Pomier‐Layrargues G . Usefulness of transjugular intrahepatic portosystemic shunt in the management of bleeding ectopic varices in cirrhotic patients. Cardiovasc Radiol 2006; 29: 216–219. 10.1007/s00270-004-0346-4 16284702

[24] Ohta M , Yasumori K , Saku M , Saitsu H , Muranaka T , Yoshida K . Successful treatment of bleeding duodenal varices by balloon‐occluded retrograde transvenous obliteration: A transjugular venous approach. Surgery 1999; 126: 581–583. 10486613

[25] Matsui S , Kudo M , Ichikawa T , Okada M , Miyabe Y . The clinical characteristics, endoscopic treatment, and prognosis for patients presenting with duodenal varices. Hepatogastroenterology 2008; 55: 959–962. 18705307

[26] Selçuk H , Boyvat F , Eren S *et al.* Duodenal varices as an unusual cause of gastrointestinal bleeding due to portal hypertension: A case report. Turk J Gastroenterol 2004; 15: 104–107. 15334321

[27] Sonomura T , Horihata K , Yamahara K *et al.* Ruptured duodenal varices successfully treated with balloon‐occluded retrograde transvenous obliteration: Usefulness of microcatheters. AJR Am J Roentgenol 2003; 181: 725–727. 12933468 10.2214/ajr.181.3.1810725

[28] Pavel V , Scharf G , Mester P *et al.* Partial splenic embolization as a rescue and emergency treatment for portal hypertension and gastroesophageal variceal hemorrhage. BMC Gastroenterol 2023; 23: 180. 37226088 10.1186/s12876-023-02808-1 PMC10207732

[29] Matsubara Y , Tsuboi A , Hirata I *et al.* Predictive factors of portal hypertensive enteropathy exacerbations based on long‐term outcomes. BMC Gastroenterol 2024; 24: 287. 39187770 10.1186/s12876-024-03377-7 PMC11346274

[30] Anand R , Ali SE , Raissi D , Frandah WM . Duodenal variceal bleeding with large spontaneous portosystemic shunt treated with transjugular intrahepatic portosystemic shunt and embolization: A case report. World J Radiol 2019; 11: 110–115. 31523400 10.4329/wjr.v11.i8.110 PMC6715580

