# Supplementary figures and images for: Analysis of the treatment outcome of duodenal varices: A retrospective case series of 15 patients from a single institution
Source: DEN Open. 2025 Apr 16;5(1):e70119. doi: 10.1002/deo2.70119 (PMC12003208; doi:10.1002/deo2.70119)

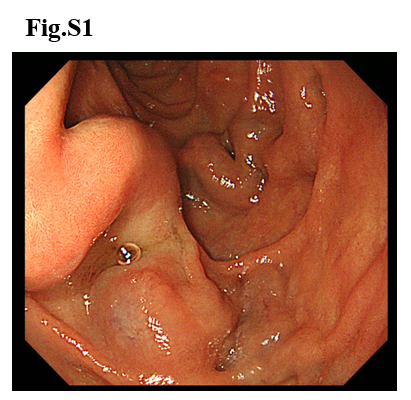

Supplement: Supplementary file 1 — Fig S1: A 56‐year‐old man with alcoholic cirrhosis was pointed out a duodenal varix in the descending part of the duodenum. [file DEO2-5-e70119-s001.png]

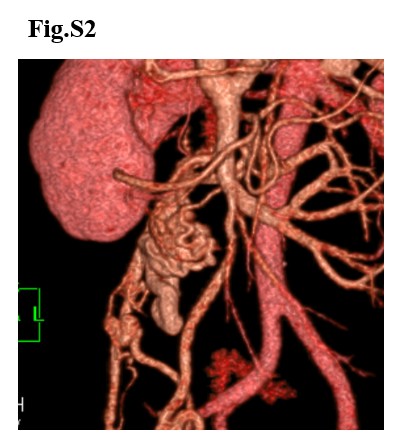

Supplement: Supplementary file 2 — Fig S2: Contrast‐enhanced computed tomography scanning showed that a small branch of the superior mesenteric vein was the major feeding vein of the duodenal varices, and the right testicular vein was the drainage vein. [file DEO2-5-e70119-s003.png]

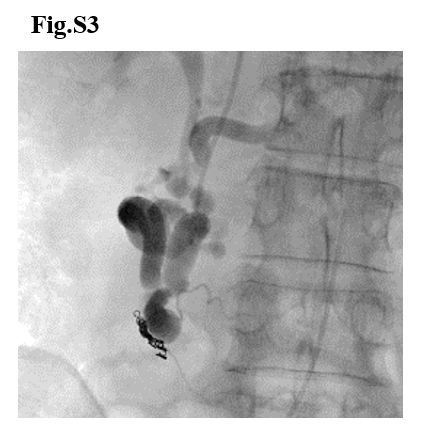

Supplement: Supplementary file 3 — Fig S3: During the balloon‐occluded retrograde transvenous obliteration, multiple drainage veins were detected that required coil embolization. [file DEO2-5-e70119-s002.png]

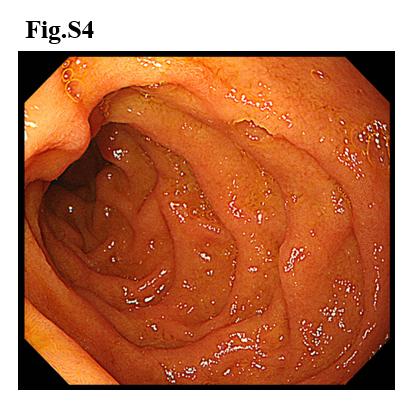

Supplement: Supplementary file 4 — Fig S4: The complete regression of the duodenal varices was confirmed after 7 months of the treatment. [file DEO2-5-e70119-s004.png]
